# Heterogeneous OPAT regimens within and across infection diagnoses: Day-level medication use patterns among 2072 OPAT patients

**DOI:** 10.1017/ash.2023.278

**Published:** 2023-09-29

**Authors:** Madison Ponder, Renae Boerneke, Asher Schranz, Michael Swartwood, Claire Farel, Alan Kinlaw

## Abstract

**Background:** Patients receiving outpatient parenteral antimicrobial therapy (OPAT) are often medically complex and require carefully tailored treatments to address severe and often concomitant infections. Our objective was to illustrate the heterogeneity in antimicrobials used for patients in OPAT, within and across infection diagnosis groups. **Methods:** We abstracted electronic health record data regarding day-level treatment into a registry of 2,358 OPAT courses (n = 2,072 unique patients) treated in the University of North Carolina Medical Center OPAT program during 2015–2022 (total, 11,861 person weeks; average, 7 OPAT weeks per patient). We classified infection diagnoses into 10 hierarchical or mutually exclusive categories (eg, bacteremia only, diabetic foot infection (DFI) only, osteomyelitis only) (Fig., vertical axes). Accounting for 64 antimicrobial medications and 520 cocktails administered for at least 1 patient day in our OPAT registry, we also defined 18 hierarchical or mutually exclusive classifications of treatment (eg, “daptomycin alone” or “daptomycin and any other antibiotic(s)” (Fig. key). We conducted 2 stratified analyses to describe the heterogeneity across infection diagnoses with respect (1) to medications used at OPAT initiation (patient as unit of analysis) and (2) to medications used throughout OPAT (person time as unit of analysis, allowing for differential OPAT course to other treatment classifications during follow-up). We present stacked bar charts to visualize the intersection between infection diagnosis and treatment group. **Results:** Among patients in this OPAT registry, 34.6% had osteomyelitis and/or DFI, 4.8% had bacteremia, and 44.6% had multiple infections (Fig. 1). The most common medications in initial OPAT regimens were vancomycin (30.8% of OPAT patients), ceftriaxone (15.0%), and daptomycin (10.9%). We observed overall similarity between the distribution of treatment groups at initiation compared to cumulative person-time during the OPAT course (Figs. 1 and 2). However, we observed heterogeneity in medications by infection diagnosis (Figs. 1 and 2); for example, vancomycin was used in 39% of osteomyelitis cases but only 14% for endocarditis (Fig. 2). For several infection groups (eg, osteomyelitis, DFI, multiple infections, “other” single infections), no treatment classification exceeded 20% use (Figs. 1 and 2). **Conclusions:** Day-level data on medication use in this monitored registry of patients provided evidence of heterogeneity in the types of medications used throughout treatment in OPAT, which varies within and across infection diagnoses. These data highlight the need for multilayered ascertainment of medication exposure in this medically complex patient population to inform surveillance for adverse effects and guide comparative effectiveness research for postdischarge antibiotic treatment.

**Figure:**
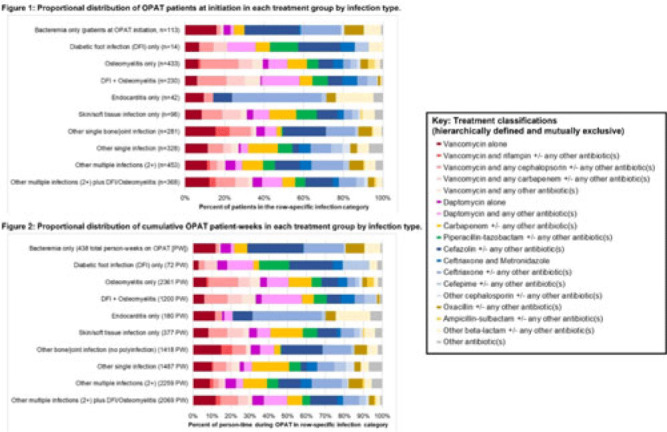

**Disclosures:** None

